# The complete mitochondrial genome of a field cricket *Turanogryllus eous* (Insecta: Orthoptera)

**DOI:** 10.1080/23802359.2019.1687034

**Published:** 2019-11-06

**Authors:** Chuan Ma, Licui Zhang, Jianke Li

**Affiliations:** Institute of Apicultural Research/Key Laboratory of Pollinating Insect Biology, Ministry of Agriculture, Chinese Academy of Agricultural Sciences, Beijing, China

**Keywords:** Mitochondrial genome, *Turanogryllus eous*, gene rearrangement

## Abstract

The complete mitochondrial genome (mitogenome) of a field cricket *Turanogryllus eous* Bey-Bienko, 1956 was determined using next-generation sequencing. The mitogenome was 16,045 bp in length comprising 13 protein-coding genes (PCGs), 22 transfer RNA (tRNA) genes, two ribosomal RNA (rRNA) genes, and a control region. Relative to the ancestral insect gene order, *T*. *eous* possessed an inversion of *trnN*-*trnS1*-*trnE*. The control region contained 3.4 tandem copies of a 194-bp sequence. Phylogenetic analysis supported that *T*. *eous* was sister to the clade comprising *Teleogryllus* and *Velarifictorus*. This study provides essential genetic information for genetic diversity analysis of *T*. *eous*.

*Turanogryllus eous* Bey-Bienko, 1956 is a field cricket species mainly distributed in eastern China and the Korean peninsula (Kim 2012; Cigliano et al. 2019). Due to their calling songs, males of *T*. *eous* are collected and traded as one of the singing pets in China. It is suggested that such collection results in a reduction in the ranges and populations of singing insects (Jin and Yen 1998). Investigation into the genetic diversity of *T*. *eous* is hampered due to the lack of molecular data. As an important genetic marker, mitogenome is extensively used in insect population genetics (Cameron 2014). In this study, the complete mitogenome of *T*. *eous* was determined using next-generation sequencing.

Samples of *T*. *eous* were obtained from a pet market in Shanghai, China (31.188°N, 121.437°E). Species identification was based on morphological characteristics. They were preserved in 100% ethanol and deposited with an accession number TE47 in the entomological specimen room in the Institute of Apicultural Research, Chinese Academy of Agricultural Sciences. Genomic DNA was extracted from a hind leg using a DNeasy Blood & Tissue kit (Qiagen). Indexed libraries were prepared and sequenced on HiSeq 2500 (Illumina Inc.) following the manufacturer's protocol to obtain 150-bp paired-end reads. The full mitogenome was assembled as described by Ma and Li (2018). Mitogenome annotation was conducted on the MITOS webserver (Bernt et al. 2013) followed by manual refinement. To validate tandem repeat sequences in the non-coding control region, a 2.4-kb fragment spanning the whole control region and flanking genes was amplified via PCR followed by Sanger sequencing. The final mitogenome sequence was deposited in GenBank (accession number MK656322). Tandem repeats were recognized using the online Tandem Repeats Finder (Benson 1999). Phylogeny of the family Gryllidae was reconstructed based on nucleotide sequences of the 13 PCGs. The partitioned models selected by PartitionFinder v2.1.1 (Lanfear et al. 2017) were used in MrBayes v3.2 (Ronquist et al. 2012) for the Bayesian inference.

The entire mitogenome of *T*. *eous* was a circular molecule of 16,045 bp in length with an A + T-biased nucleotide composition (40.06% A, 31.01% T, 19.37% C, and 9.55% G). The mitogenome consisted of 37 genes (two rRNA genes, 22 tRNA genes, and 13 PCGs), a 1,292-bp non-coding control region, and multiple short intergenic spacers. Compared with the ancestral gene arrangement of insects, *T*. *eous* possessed an inversion of the *trnN*-*trnS1*-*trnE* to *trnE*-*trnS1*-*trnN*, which was commonly found in all sequenced mitogenomes of the family Gryllidae. Most PCGs started with typical ATN codons (six with ATG, three with ATT, one with ATA, and one with ATC), whereas *cox1* and *nad1* started with non-canonical TCG and TTG, respectively, which were verified to act as start codons in transcript studies (Besansky et al. 2006; Stewart and Beckenbach 2009; Ma and Li 2018). Five PCGs ended with complete stop codons (TAA or TAG), while the others were proposed to utilize partial stop condons (T or TA) immediately preceeding a downstream gene. A 194-bp sequence was found to tandemly repeat 3.4 times in the control region. These repeats shared a high sequence identity (>98.8%). In addition, nine intergenic spacers ranging in size from 2 to 24 bp and seven overlaps (1 − 8 bp) between adjacent genes were identified. The phylogenetic tree strongly supported subfamilial monophyly of the family Gryllidae (Figure 1). Within the subfamily Gryllinae, the newly sequenced *T*. *eous* was sister to the clade consisting of *Teleogryllus* and *Velarifictorus*. The monophyly of the tribe Gryllini represented by *Teleogryllus* and *Loxoblemmus* was not supported due to the inclusion of *T*. *eous* (Turanogryllini) and *Velarifictorus hemelytrus* (Modicogryllini).

**Figure 1.**
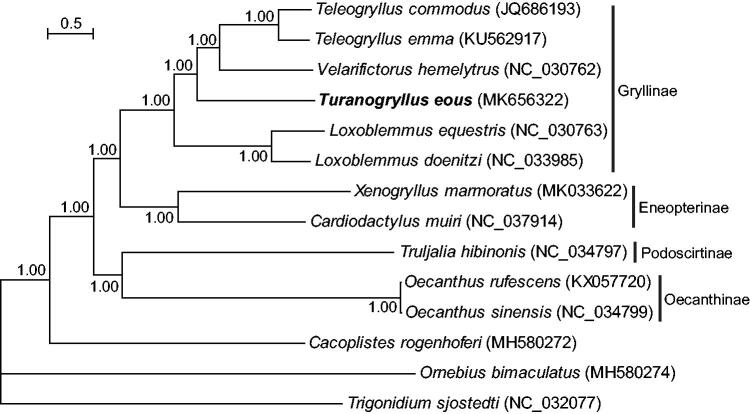
The phylogenetic tree of the family Gryllidae inferred from 13 protein-coding genes. Bayesian posterior probability values are indicated at nodes. GenBank accession numbers for each taxon are provided in parenthesis.
